# Abstract of Proceedings of the Lake Erie Dental Association

**Published:** 1871-08

**Authors:** 


### ARTICLE II.

### *Abstract of Proceedings of the Lake Erie Dental Association.*

This Association met in Meadville, Pa., June 6th, at 10.30 A. M., Dr. G. B. McDonneld, President, in the chair. In the discussion during the sessions Dr. Robbins said that the chemical theory of decay is the accepted one, the old theory being that decay proceeded from within outward; but now that it proceeds from without inward; thinks we have a combination of chemical and thermal agents causing decay by the decomposition of food lodged about the teeth. So the limestone of houses in eastern Pennsylvania crumble and disintegrate under the thermal and chemical influences of the atmosphere. Teeth resist chemical action better than do the other animal tissues which are constantly changing; but the teeth have not the same power of repair which belongs to the other tissues. In order to understand the chemistry of the teeth we must understand general chemistry. Lime salts should be administered through the food, as in brown bread, lime water in white bread, and directly, as hyphosphite of lime, &c.

Dr. Ansart mentioned that in South America a Chinese Coolie who would eat no fruit but lived on meat, bread and rice, had excellent teeth, while the other Coolies, who eat much acid fruit, had crumbling teeth, like those of the natives, yet the enamel in the first case was not so thick as that of the natives.

Dr. Allen had lived in the South Sea Islands; but never known a native to have the toothache, but where he was there were few acid fruits. The health of these islanders was generally good; they had no fevers, but had pulmonary diseases; they took great delight in bathing. Among the Indians of Michigan he found many bad teeth, but they lived much as the white men did.

Dr. Wallace had lived in Valparaiso and found the teeth of the South Americans all more or less decayed. The half breeds were much afflicted; came to the conclusion that the direct cause was a hot drink called Matte. The front teeth were most affected. Those who used improper food had a different decay; those articles which had sulphur in them formed sulphurous acid, which, taking oxygen from the atmosphere, formed sulphuric acid, and this produced decay.

Dr. G. W. Nelson read an essay on the "Management of Children's Teeth.

Dr. Carroll said we never should extract the deciduous teeth before the time of the eruption of the permanent teeth if it is possible to avoid it. Would treat exposed nerves and fill as in permanent teeth. For filling these temporary teeth he likes tin, which can be put in quickly; after tin Hill's stopping, which must be renewed occasionally. Has extracted before the time for the eruption of the permanent teeth when there are well defined cases of alveolar abscess; thinks there is a tendency to contraction of the jaw on the premature extraction of deciduous teeth.

Dr. G. Elliott finds great difficulty in using gold or tin in deciduous teeth; thinks in some cases amalgam well prepared makes as good a filling for deciduous teeth as we can get; does not advocate the general use of amalgam; uses oxy-chloride of zinc most.

Dr. Ansart never uses amalgam in children's teeth; prefers Hill's stopping and Guillois' cement.

Dr. Wallace—One condition of deciduous teeth is where the permanent teeth appear before the deciduous teeth are properly disposed of. Permanent teeth come in a wrong position.

Dr. Robbins—Management of children's teeth includes the permanent teeth. Six year molar is usually the first tooth coming under the dentist's care. Parents often think it a temporary tooth and neglect it. We should treat and preserve this molar as well as all other permanent teeth; thinks that the shortening of the jaw and change of facial angle of the Anglo Saxon race is caused by the early extraction of permanent teeth. Nature's model is the best; we cannot restore the contour of the face after the extraction of cuspids; should avoid amalgam if possible, but would use it rather than extract.

Dr. C. D. Elliott has better success with cement plombe than any other plastic filling. Gave a case of nine months standing, which is still good.

Dr. McDonneld mentions an oxy-chloride of zinc filling, which has lasted two years. The density of *os-artificiel* depends on the method of manipulating it.

Dr. Ansart called for information concerning the lancing of children's gums.

Dr. Griffin—When the gums are tumified, inflammation high, and children almost in convulsions, would not hesitate a moment to lance. Thinks there is much virtue in Homœopathic medicines in these cases.

Dr. Ansart would invariably lance when the membrane is tense and the inflammation is high, passing a sharp lance directly down to the tooth on the line of the cutting edge.

Dr. Dunn said that cicatrices yielded more readily to pressure than normal tissues, so that lancing did not retard the eruption of the tooth.

Dr. G. Elliott thought that more pain proceeded from the tension than from congestion.

Dr. Wallace said that the congestion was caused by pressure. The arrested blood corpuscles pressing on nerve tissues cause pain. This condition was relieved by lancing. Discussion closed.

Dr. Carroll then read his essay on "Alveolar Abscess," describing its various forms and the treatment required. His rule for applying local remedies, was to choose that most agreeable to the patient, whether hot or cold. Discussion followed. To illustrate, Dr. Carroll took the case of a central incisor. Would inject an escharotic. When all inflammation was subdued and the fistula healed, would fill the root solidly, and, if the trouble is renewed, would open through the alveolus, and remove the sac mechanically.

Dr. Templeton has had more success with this method than with any other, but fills before treating. Treatment through the canal is not effective unless we can force the creosote through the fistulous opening. After breaking up the sac, applies creosote. Some times uses an anæsthetic before operating. If this treatment fails, it is because we do not reach the spot when opening through the gum.

Dr. McDonnald has been treating abscess for six years, and is confident that he has cured 99 in 100 cases in the superior maxilla. In the inferior maxilla is not so successful. Injects an escharotic, usually creosote, through the pulp canal. Almost all cases of failure are due to an impaired condition of the blood, or as in the inferior molars, to the difficulty of reaching the sac. The escharotic changes conditions and destroys life, the absorbents then carry off the effete matter if the person is healthy.

Dr Robbins remembers when it was considered unprofessional to treat alveolar abscess and when he was called a fool, by professional men for attempting it We must use the term cure for want of a better one In some cases apparently healthy, it is difficult to decide why we do not cure. In others a constitutional taint, like syphilis is easily detected. When the foramen of the canal is small and there is no fistula, must make an opening through thorugh the gum. The best instrument for this purpose is a sharp Gates' Drill which goes through the process easily and brings out the cuttings, leaving a smooth round opening. When, after being apparently cured, inflammation supervenes,may often arrest it by counter irritannts upon the gums. The action of Iodide of Potassium in these cases is illustrated in Dr. Watts' Chemical Essay.s The Iodine and Potassium are like coupled dogs harmless till they reach the point of obstruction, when they are released. The Iodine goes off with oxygen as Iodic Acid, while the caustic potash dis-olves the obstructions.

Dr. Geo. Elliott considers the treatment of alveolor abscess in the same light as other surgical operations. After breaking down the sac, introduce remedies which resolve the material and prepare it to pass out, then keep the fistula open till the cavity is filled up by healthy granulations proceeding from the bottom to the top.

Dr. Herrick read his essay on "Mechanical Dentistry."

Discussion—Dr. Templeton. To obviate air holes in plaster models, soaks the impression half an hour in water, having thinly coated it with varnish to give color, and pours without oiling.

Dr. Pierce uses analine in the water to color impressions, and uses Silex for parting.

Dr. C. D. Elliott when taking impressions, uses the plaster thin if the palate is spongy, so as not to push away the soft parts. If the parts are firm mixes the plaster thicker. When taking impressions for partial sets, if the natural teeth are ill shaped, oils the impression cup before mixing the plaster. After taking the impression removes the cup first and splits the plaster before removing it.

Dr. McDonneld thinks that the expansion of impression results from having too thick a body of plaster, to be remedied by using wax first and over that a thin coating of plaster. After taking the impression puts in water to prevent heating which causes expansion.

Dr. Dunn thinks expansion takes place in the vulcanizer.

Dr. Templeton—To get a good articulating bite directs the patient to sit upright and stretch the muscles of the neck before closing the jaws.

Dr. Ansart—For partial sets takes impressions before extracting roots, &c., and carves out the plaster—getting good results.

Dr. C. D. Elliott has put up one set of teeth on the Hyatt Perkins base. If it will witstand the fluids of the mouth, and wears well, it will supercede rubber. Put up the case in 1½ hours.

Dr. Wallace describes the method of using pyroxyline, which he prefers to rose pearl. It is composed principally of gun cotton and ether submitted to heavy pressure which presses out the ether. For soldering it, filings of the same material itself reduced to a pulp by ether, are used.

Dr. Robbins—The Hyatt Perkins base contains camphor. Is afraid of the effect of camphor on the mucous membrane. This base is inflammmable as is the case with all collodion bases.

Dr. Tenney has cast Aluminum plates. The process requires great nicety of manipulation to obviate the effects of shrinkage when cooling. After casting, must jar the flask and plunge two thirds of it into wet sand where it must be rotated continually, the plunger being pressed hard against the mouth of the flask, the upper part of which must be kept warmer than the lower. Aluminum melts at about 1500° gets better fitting plates of Aluminum than of rubber.

Dr. Hawxhurst swages thin plate of Aluminum on type metal dies, the shrinkage of which is about right. Fastens the teeth with Watt's and Williams' metal, columbium. Drills holes in the alveolar ridge, countersinks and fills them with pure wax. Proceeds as for a rubber case, using powdered pumice in the plaster to prevent shrinkage; heats the flask almost to the melting point of the metal; then having the metal as nearly as possible at the melting point, pours the metal and jars the flask.

Dr. Tenney suggested the use of parafine instead of wax to fill the countersinks, as it will disappear entirely when heated.

Dr. Robbins thinks the safest and best way to use rubber is an attachment to gold plate. Passes a continuous rim of gold around inside the teeth; does not perforate the plates but solders gold loops to the plate to hold the rubber. This makes the best denture, being cleanly and strong and the thermal changes are good.

Dr. Harrison gave his method of resuscitating patients who are sinking under the use of chloroform. He passes the feather end of an uncut quill down the throat and gives it a rotary motion. The patient will revive instantly, there being sympathetic connection between the glottis and the heart. Has always succeeded in this way.

Dr. Robbins—When electricity is applied directly to the tooth, it is more unpleasant than when applied to the gum—Have more direct and concentrated current when the pole is applied to ganglia than when it is held in the hand.

When applying the rubber dam it is often convenient to use the string in a half hitch. Another way to hold rubber in place, is by a wedge pressed between the teeth by a perforated forceps. The rubber dam is the best protection to the dentist against fœted breath of patients. Dr. Butler, of Cleveland always applies the rubber dam before beginning to excavate; can remove the debris with a dry syringe. To avoid danger of periostitis and abscess from the forcing of fluids through the foramen of the apex of the root, when filling, use the hot air syringe, which will dry out the root canal better than anything else.

For trimming down approximal fillings, finds a sickle shaped, knife-edged instrument very useful. Rouge cloth gives a good finish. Chalk gives the best final polish.

After filling on the grinding surface, always cuts down the gold until the teeth can close without striking the gold, before allowing the patient to leave the office.

Advocates the use of steel as a material for mallets. Likes nickle plating to prevent rusting. Uses thin shellac varnish for the same purpose.

Dr. Hawxhurst suggested the use of weak collodion to prevent rusting of instruments, the weaker the better, provided a film is formed.

Dr. Ansart says that Dr. Harvey of Buffalo, when applying the rubber dam over lower molars, in difficult cases, attaches a small piece of leather to the string and places it so that the leather rests against both the rubber and the tooth, and prevents the string and rubber from slipping.

Dr. Ansart described Bornwell's electric mallet also an instrument which can be attached to any plugger, and by means of which gold can be used in long strips.

Dr. Hawxhurst said the principle use of Green's pneumatic engine was for trimming down gold and cutting retaining pits—the pneumatic plugger was not so successful.

Dr. McDonneld, after filling approximal cavities, tries to leave no space between the teeth after the operation is finished; otherwise food will lodge between the teeth and be very troublesome.

Dr. Hawxhurst spoke of teeth worn down by occlusion, and asked for a remedy.

Dr. McDonneld always fills such teeth with gold.

Dr. Wallace had such a case where the superior incisors were worn away, and the inferior incisors beveled very thin and sharp. He built up the bicuspids, and cut down the inferior incisors 1–16 of an inch.

